# Construction and validation of the Basic Scale of Entrepreneurial Competencies for the Secondary Education level. A study conducted in Spain

**DOI:** 10.1371/journal.pone.0249903

**Published:** 2021-04-15

**Authors:** Antonio Ramón Cárdenas-Gutiérrez, Antonio Bernal-Guerrero, Elisabet Montoro-Fernández

**Affiliations:** Department of Theory and History of Education and Social Pedagogy, Faculty of Education Sciences, Seville University, Seville, Spain; Aalborg University, DENMARK

## Abstract

**Introduction:**

The impact of entrepreneurship training on the levels of compulsory education has been weak until now. Complementarily to the demand of greater effectiveness in entrepreneurship education, it is a priority to make scientifically verified instruments available to provide useful information about the achievement of the competences needed for the development of entrepreneurial capacities. Our research is focused on the design and assessment of entrepreneurship competency, tackling one the dimension concerning business skills or competences. Specifically, the aim of the study consists in the development, validation and reliability of an instrument, intended for secondary education, created with the purpose of detecting the strictly indispensable entrepreneurial competencies in the basic training of the business profile, the Basic Scale of Entrepreneurial Competencies (BSEC).

**Methods:**

The research was developed in three phases via qualitative and quantitative methods. In the development phase the items were generated and the dimensions and components of entrepreneurship competency were identified. Also, the content and face validity were carried out, where experts (n = 48) and students (n = 24) took part. In the recruitment phase a multi-stage sampling stratified by conglomerates was performed, obtaining a sample of 1440 students, aged between 11 and 17 years old (M = 14.6, SD = 1.597) and a composition by sex of 679 girls and 761 boys. Construct validity was evaluated in the assessment stage through factor analysis (EFA and CFA). Later, the reliability was studied via the Cronbach´s *α* coefficient and the stability and reproducibility over time with the test-restest technique. Finally, the convergent and divergent validity were evaluated through the average variance extracted (AVE), the composite reliability (CR) and the square root of the AVE.

**Results:**

44 items were developed in the phase of generating items. After carrying out the validity of the content, there were 14 items with acceptable values in the Content Validity Ratio (CVR.89) and in the Content Validity Index (CVI.92). During the validation of the scale, the results of the exploratory and confirmatory factor analysis confirmed a first-order trifactorial structure and a second-order factor. The scale’s stability was appropriate, having an ICC = .92. The convergent validity results with Composite Reliability (CR) scores > 0.7 and the Average Variance Extracted (AVE) >. 0.50, along with the square root values of the AVE greater than the correlations between the other constructs show us important evidence of the validity of the Scale. The structure of the BSEC is made up of 13 items and three domains: Operations and Marketing Competencies (OMC), Competencies in Socio-Business and Legal Organization (CSBLO) and Economic-Financial Competencies (EFC).

**Conclusions:**

The results of the research reflect its validity and reliability. This Scale has an evident usefulness for the training and assessment of entrepreneurship competence. Specifically, it is efficient for the valuation of entrepreneurial competencies in adolescent students in the stage prior to their incorporation into the work environment or their integration into the itineraries leading to higher education levels.

## Introduction

The new economy is directly linked to the relevance of knowledge. The improvement of quality of life is more related to learning than to the effectiveness of resource allocation, the traditional concern of economists. As Joseph E. Stiglitz and Bruce C. Greenwald [1] have expressed, resource allocation is a slow process if we compare it with the speed with which we can reduce knowledge gaps. Due to its association with innovation, entrepreneurship and economy, human capital has therefore become a focus of the utmost attention [2–5]. In fact, the challenges currently posed to the development of the economy require training to tackle the face-to-face global transformations in the business field, to compete in the business world and to remain open to continuous renovation in the face of constant demanding changes. This training requires specific knowledge, certain attitudes and concrete competences or precise behavior features. This can be seen in Order ECD/65/2015 [6], where entrepreneurial competence is defined as a feeling of initiative and entrepreneurial spirit, as a competence which favors the transformation of ideas into acts. This becomes specific in the capacity to select, plan and manage knowledge, skills and attitudes on one’s own terms to attain a particular purpose. More concretely, and in relation with our study aim, this Order indicates that entrepreneurial competencies are related with the organization and functioning of firms through business planning, organization and management, without ignoring the ethical aspects of the business activity. In this way, entrepreneurial competencies would be defined within a global approach of the business competence and would be linked to certain business actions, such as management, organization, operations, marketing, financing or entrepreneurial strategy [7].

The new firm’s ecosystem, made up of incubators, intermediaries, accelerators, assessors, etc., tends to recognize the difference between the two phases characterized by their particular activities: exploration and exploitation. This distinction highlights the need of knowledge prior to beginning business creation per se, trying to minimize the gap between learning and innovation [8–10]. Moreover, it is evident that entrepreneurs’ competences greatly affect their decisionmaking, which also contributes to the business efficiency, as has been stressed by some recent studies [11–13].

There seems to be an international consensus concerning the relevance conceded to entrepreneurship as one of the main economic activities in the current context [14, 15]. The business spirit fosters self-employment in the new generations, raises their level of employability and nurtures the fundamental social skills for professional development [16, 17]. Hence, immersive proposals have been done based on real problems of firms with the aim of training in entrepreneurial competences [18, 19]. In general, it appears to be deduced from the majority of studies performed that specific business education programs maintain a positive link with entrepreneurship, affecting the very choice of becoming an entrepreneur, as well as later business success [20–22].

Nonetheless, although the global consensus about the importance of entrepreneurial culture for economic and human development has for a long time been integrated into the political plans of the majority of countries, it can be considered that the influence of political guidelines on the need for training in entrepreneurship in compulsory education (Middle School and High School), in the basic stages of formal education, has until now been generally weak [23]. It is also true that education and training policies should reflect a greater theoretical and practical solidity, showing in the best way possible the nature and the development of business skills and offering a more consistent training panorama to the whole educational system [24]. This is particularly so in the compulsory education levels as a basic shaping of the entrepreneurial identity that entrepreneurs will later need [25, 26]. This necessary political orientation especially includes the professionals of entrepreneurship education [27]. The considerable volume of research works carried out, although still insufficient in the basic levels of education [28], shall constitute a determinant material for a better demarcation of the school policies and practices linked to business education.

In this sense, as an indispensable supplement of the generalized demand for greater effectiveness in entrepreneurship education, it is fundamental to dispose of validated and operational instruments capable of providing useful information concerning the achievement of the competences needed for the development of entrepreneurship. Nevertheless, the adoption of diverse perspectives about business competence causes different problems concerning the valuation of the skills needed, giving rise to the convergence, at times confusing, of different factors of a social, political and economic character [29]. Though we could state that competence, in general, alludes to the virtue or status of being an entrepreneur, competency refers to some specific attribute possessed by someone within a set of related competences. Linked with the context, competences delimit an essential perspective of entrepreneurship education [30]. Consequently, research on the design and evaluation of entrepreneurial competency is an indispensable area for the efficient development of the training of entrepreneurs.

But, however, there are very few questionnaires which go thoroughly into this training dimension of entrepreneurship. Particularly, one notes a significant gap in the levels of compulsory education. The innovative behavior of employees has been explored through an evaluative scale of the intrapreneurial competences [31] (promotion of opportunities, proactiveness, flexibility, impetus and risk taking) with the idea of furthering the line of a robust model of intrapreneurial competences in the framework of firms, singularly in its role of a diagnosis test to enhance the development of workers in areas associated with the generation and innovation of enterprisess [31].

Likewise, within the theoretical perspective of professional construction, entrepreneurship has been analyzed as an adaptive vocational behavior driven by a person’s capacity of self-regulation to prosper in the framework of their professional career. The Career Adapt-Abilities Scale (CAAS) has been validated in a sample of business students, showing evidence of its psychometric properties and offering elements for the comprehension of the successful adaptation in the context of business careers. It is positively associated with business intentions and with prior experience in family businesses [32].

There exists some psychometric research related to a specific element of the set of entrepreneurial competences. Thus, the recognition of business opportunities is one of the outstanding skills of entrepreneurs and, therefore, needs attention in business education. Starting out from existing models to measure the creativity and the recognition of opportunities, the questionnaire Perception of Opportunity Recognition Ability (PORA) has been created, showing an acceptable internal consistency [33].

In some countries representative of a great part of the global population, such as India, studies have been performed via the creation of questionnaires capable of diagnosing the common start-up problems and the success factors which contribute to the entrepreneurial process according to differences of sex [34], among adults interested in entrepreneurship and business development.

We observe that the assessment of entrepreneurship competency has a long track record and is mainly oriented toward adults in pre-business or business training contexts [35–41]. In this sense, a lack of assessment instruments related with entrepreneurship competency in the compulsory education stages has been detected. This research work proposes to study the validity and reliability of an instrument created with the aim of palliating the deficiency identified: the Basic Scale of Entrepreneurial Competencies (BSEC) intended for adolescents in the Secondary Education stage.

## Materials and methods

This research is part of the project “Educating in Entrepreneurship: Evaluating Programs for the Training of Entrepreneurial Identity in Compulsory Education”, financed within the State Plan of Excellence of the Government of Spain (Reference: EDU2013-42936-P). Among other results stemming from this R+D+i (Research, development and innovation) project, initial research has emerged concerning the study of the validity and the reliability of the Basic Business Knowledge Scale for secondary education students [42]. In parallel to this first study about business knowledge, and following the methodological orientations proposed in it, this second study has been developed concerning the psychometric properties of the Basic Scale of Entrepreneurial Competencies (BSEC).

### Study design

We have carried out a transversal study with a design of mixed three-phase methods [43]: Phase 1) development and construction of the Basic Scale of Entrepreneurial Competencies (BSEC); phase 2) recruitment of the sample; and phase 3) assessment of the reliability and validation of the Scale (Table 1).

**Table 1:** Construction process and chronological axis of the research design.

**Phase 1: Development and construction of the BSEC**	
**Sub-phases**	**Dates and participants**
**1) Theoretical foundations and generation of items**	From January 7th. to March 29th. 2018
Bibliographic review in databases	Experts (n = 4)
Interviews of experts	Expert teachers (n = 8)
Interviews of students	Students (n = 12)
Experts´judgment	Experts from university-educational centers (n = 16)
**2) Content validity**	From April 2nd. to April 30th. 2018
Assessment of experts about formal aspects of the items	Experts (n = 10)
Assessment of experts CVR and CVI	Experts (n = 10)
**3) Face validity**	May 11th. 2018
Focus group	Students (n = 12)
**Phase 2: Recruitment of the sample**	
**Sub-phases**	**Dates and participants**
**1) Recruitment and data collection**	From April 5th. to July 15th. 2018
Characteristics of the sample	Sample (n = 1440) 679 girls and 761 boys
**Phase 3: Evaluation of the validity and reliability**	
**Sub-phases**	**Dates and participants**
**1) Construct validity**	From July 18th. to July 22th. 2018
Exploratory factor analysis	Sub-sample (*n*_1_ = 720)
Confirmatory factor analysis	Sub-sample (*n*_2_ = 720)
**2) Reliability**	From July 25th. to August 18th. 2018
Cronbach Alpha	
Test-retest time stability	Sample (n = 50)
**3) External Validity**	From August 20th. to August 25th. 2018
Convergent validity	
Divergent validity	

#### Phase 1: Development of the questionnaire

*1) Theoretical foundations and generation of items*. To implement this sub-phase we have developed the PRISMA-P protocol [44] (S1 Checklist), although applying only 4 items corresponding to the sections of methods and results, as it is an educational bibliometric review [44, 45], with the aim of generating the composition and dimensionality of the basic business competences, we carried out a systematic bibliometric review in January 2018 in the main databases (WOS, ERIC and Scopus), applying the following search strategy with the protocol: “Entrepreneur* competencies” OR “Entrepreneur* competency”. The bibliographic search was limited to scientific articles from January 1st. 2000 to December 31st. 2017. Later, four university field experts with broad and acknowledged experience and scientific careers in business education valued the potential articles. The articles which had the following keywords either in their titles or their abstracts were selected: a) competency/ies; b) entrepreneurship education and entrepreneurial education or business education; c) compulsory education; d) Middle School and High School; and e) evaluation and assessment. The eligibility requirements were: a) Type of sample studied, including studies centered on the compulsory and post-compulsory stages of formal education; and b) typology of study, including theoretical, empirical and qualitative research works. As an exclusion criterion, we adopt that the article must be classified as “not relevant” (studies related to non-formal education) by at least two experts. Having selected the articles, they were submitted to a content analysis through an inductive coding [46]. Lastly, to find out the degree of agreement between the experts the Fleiss’ kappa index was used [47], since it is valid to measure the agreement with three or more experts [48].

Later, between February 1st. and March 29th. 2018, two series of detailed interviews were done via a convenience sampling (S1 and S2 Files). On the one hand, there were 8 teachers, experts in business education in Compulsory Secondary Education centers, being selected two teachers per course of this stage. The choice of these experts was done through the criterion of participation as coordinators of business education programs during 3 academic courses. On the other hand, 12 students from Compulsory Secondary Education centers were interviewed with the criterion of having taken part in business education programs during the 2016-17 academic course. All the interviews lasted between 35 and 50 minutes and were analyzed via an analysis of their content with the NVivo 11 Plus software. The experts’ judgment technique was applied to attain a high degree of validity of the scripts of the interviews, eight of them from the university area and another eight from educational centers [49, 50]. All of them had a broad professional and academic experience in the area of entrepreneurship education. In order to attaining a high consensus among the experts and reducing the variability of their valuations, a criterion of surpassing 80% of concordance in the indications given by them in the two evaluation sessions was established.

*2) Content validity*. Two sub-phases were performed. In the first one, 10 experts (two for each of the following areas: business education in compulsory education, firm organizational and legal management, marketing, economics and assessment of instruments) carried out an assessment related with the formal and written aspects of each of the 44 items developed by the research team. The items were revised with the criterion of at least one of the experts explicitly indicating the need for a revision. In the second, the Content Validity Ratio (CVR) and the Content Validity Index (CVI) were studied. To do so, another 10 specialists, two for each of the areas previously mentioned, analyzed, on the one hand, the CVR, evaluating each item with a 3-point Likert scale from non-essential to highly essential. Those items whose score was ≥.62 were kept in the instrument [51, 52]. On the other hand, the CVI was calculated for each item [53, 54] via a 4-point Likert scale from not relevant to highly relevant [55], those items which attained scores equal to or greater than.80 were selected. Later, the total CVR and CVI of the Scale were calculated.

*3) Face validity*. This sub-phase was developed through a focus group made up of 12 students chosen through a convenience sampling with similar age range characteristics to the studied sample. The selection criterion was to have participated during more than two academic courses in entrepreneurial education programs. The age of the students ranged from eleven to sixteen years old, the focus group being made up of two students per age. The duration of the focus group was 90 minutes and the Think-aloud protocols [55–60] were used as a comprehension technique to find out the ideas, suggestions and changes made by the students for each item. The focus group was guided, recorded, transcribed and analyzed with the NVivo 11 Plus program. The content analysis was done inductively via the following categories: formal problems and conceptual problems.

#### Phase 2: Recruitment and sample

*1) Recruitment*. The sample design was a multi-stage sampling stratified by conglomerates. The study population were the education centers which had taken part in at least two editions of the entrepreneurial education program. The sample selection criteria are listed below:

Criterion 1. Entrepreneurial education strategies: the Oslo Agenda [61] defines three strategies in entrepreneurial education: specific, general and isolated initiatives. Two autonomous communities were chosen with this criterion for each strategy: Andalusia and Galicia, with a specific strategy; Asturias and the Region of Murcia, with a general strategy; and Madrid and Catalonia with an isolated initiatives’ strategy [62].
Criterion 2. The selection of education centers supported by public funds which were teaching YEE (Young European Enterprise) entrepreneurial education programs in Compulsory Secondary Education. Information was gathered from the databases of the UECOE (Spanish Union of Teaching Cooperatives) and of VALNALÓN (a public firm under the authority of the Ministry of Employment, Industry and Tourism of the Government of Asturias). 16 centers fulfilled these two criteria.
Criterion 3. The YEE program should have been implemented during at least two editions. The 11 education centers of the six autonomous communities which fulfill this requirement were selected with this inclusion criterion. All the education centers accepted participating. The recruitment lasted from April 5th. to June 27th. 2018.

*2) Characteristics of the sample*. In Spain, during the academic year 2017/2018, the research was implemented in a population made up of 1,964,787 students of the ESO (Compulsory Secondary Education) stage (Ministry of Education and Professional Training). First, there were 1,473 students in the sample, but 33 questionnaires were eliminated in the data tabulation process due to the fact that many items were not answered. The sample is representative of the ESO stage (*ϵ* = 3.4% y P = 99%).

(Table 2) describes the sample’s characteristics. The composition of the final sample according to sex was 679 girls (47.2%) and 761 boys (52.8%), aged between eleven and seventeen years old (M = 14.6, SD = 1.597).

**Table 2:** Sample descriptione.

			Andalusia	Asturias	Catalonia	Madrid	Murcia Region	Galicia
Age; M			14.02	16.03	16.57	14.86	12.2	13.92
Participants; n (%)			962 (66.80%)	213 (14.80%)	60 (4.16%)	104 (7.22%)	53 (3.69%)	48 (3.33%)
Sex; n (%)	Female		519 (53.96%)	86 (40.37%)	22 (36.67%)	83 (79.81%)	31 (58.50%)	30 (62.5%)
	Male		443 (46.04%)	127 (59.63%)	38 (63.33%)	21 (20.19%)	22 (41.50%)	18 (37.5%)
Education level in ESO; n (%)	1º ESO						38 (71.69%)	
	2º ESO		392 (40.75%)					13 27.08%
	3º ESO		435 (45.22%)	78 (36.61%)		93 (89.42%)		35 (72.92%)
	4º ESO		135 (14.03%)	135 (63.39%)	60 (100%)	11 (10.58%)	15 (28.31%)	
Sex by education level in ESO; n (%)	1º ESO	F					18 (33.97%)	
		M					20 (37.73%)	
	2º ESO	F	240 (24.95%)					9 (18.75%)
		M	152 (15.80%)					4 (8.33%)
	3º ESO	F	213 (22.14%)	26 (12.20%)		38 (36.53%)		20 (41.67%)
		M	222 (23.07%)	52 (24.42%)		55 (52.89%)		15 (31.25%)
	4º ESO	F	66 (6.87%)	60 (28.17%)	48 (80%)		13 (24.52%)	
		M	69 (7.17%)	75 (35.21%)	12 (20%)	11 (10.58)	2 (3.78%)	
Centers with YEE			6	2	2	1	2	2
Centers studied with YEE, n (%)			4 (66.66%)	1 (50%)	2 (100%)	1 (100%)	1 (50%)	2 (100%)
Type of educational center	Public		NA	NA	NA	NA	NA	NA
	Private		NA	NA	NA	NA	NA	NA
	Supported with public funds		4 (100%)	1 (100%)	2 (100%)	1 (100%)	1 (100%)	1 (100%)
Educational strategies for entrepreneurship			Specific	General	Isolated initiatives	Isolated initiatives	General	Specific

Notes.

a ESO (Educación Secundaria Obligatoria-Compulsory Secondary Education).

b EJE (Empresa Joven Europea-Young European Enterprise).

c In Spain educational centres and students participate voluntarily in entrepreneurial education programs.

#### Phase 3: Assessment of the validity and reliability

*1) Descriptive analysis and discrimination indices*. The descriptive analysis of the items was done with descriptive statistics, such as the mean (μ), standard deviation (*σ*), kurtosis (K) and asymmetry. The discrimination indices (corrected item-total correlation) were calculated for each item.

*2) Construct validity*. The construct validation of the BCES was implemented through two factorial analyses (a and b). The total sample (1440) was divided into two random samples, each made up of 720 participants.

a) Exploratory Factor Analysis. An EFA was applied to the first half of the sample (720) [63]. The degree of suitability to factor analysis was done through Kaiser’s KMO, along with Bartlett’s Sphericity Test [64, 65]. The OLS (Ordinary Least Squares) method was used in the estimation of the factors and, within this, the ULS (Unweighted Least Squares) method, being [66] the most recommendable method. Having estimated the factors and to achieve a high degree of simplicity and interpretability of the factorial solution obtained, rotation was applied with the direct Oblimin technique, given that we presuppose correlations between the latent variables or factors analyzed [67]. Lastly, the factors were selected with the Parallel Analysis (AP), taking into account criteria such as eigenvalues greater than one and variance explained [68]. Also, the items with saturations less than.40 were refined.

b) Confirmatory Factor Analysis. With the second half of the sample, CFA was carried out for the purpose of checking the hypothesized model [69]. This analysis was done according to the following methodological process: 1) the ULS (Unweighted Least Squares) technique; 2) oblique rotation with the direct Oblimin criterion; and 3) the use of a set of absolute fit indicators (Standardized Root Mean Square Residual -SRMR, Goodness of Fit Index -GFI- and Root Mean Squared Error of Approximation -RMSEA) and an incremental (Tucker-Lewis Index TLI) and Comparative Fit Index -CFI-) to interpret the model extracted, as it has been studied that the χ2 statistic has a high sensitivity to the variations of the sample size [70–73]. The interpretation of the adjustment criteria is established according to the scores obtained in each indicator. First, scores equal to or greater than.96 and.95, respectively in the CFI, GFI and TLI, along with scores equal to or less than 0.5 in RMSEA, are considered a good fit. Second, scores equal to or greater than.90 in the CFI, GFI, TLI and scores below.08 in the RMSEA are estimated as an average fit. Third, scores in the CFI, GFI and TLI equal to or above.90 and the RMSEA with a score equal to or less than.10 are interpreted as a low fit. Fourth, the SRMSR with scores less than.08 are accepted as a suitable fit [70, 73].

*3) Reliability*. To evaluate the reliability, the internal consistency was analyzed with the Cronbach´s *α* coefficient of the three subscales and of the global instrument. The composite reliability (CR) was also evaluated, scores above.7 being interpreted as an appropriate internal consistency [65]. Later, the scale’s reliability was studied to find out the stability and reproducibility over time with the test-retest technique. Thus, 50 subjects filled out the scales at two different measurement moments with fourteen days between both applications [74]. Scores above.75 were considered good in the interpretation of the Intraclass Correlation Coefficient (ICC) [75, 76].

*4) External Validity*. The validity of the scale has been carried out via the analysis of the convergent and discriminant validity. The convergent validity was evaluated by means of the Composite Reliability Index (CRI) and the Average Variance Extracted (AVE), which provide information about the amount of variance explained of the construct for each of its indicators; the reference values must be ≥.70 and ≥.50, respectively [77]. The Fornell and Larcker [77] method was used to find out the discriminant validity because of its sensitivity. Here it is proposed that the discriminant validity is established when the AVE is greater than the square root of the correlations between each factor and the rest of the latent variables. The FACTOR 10.4 [78]; EQS 6.2 [79]; Smart PLS 2.0 M3 [80] and the SPSS 23.0 [81] were used of the data analysis.

### Procedure

The educational centers which had the possibility of choosing were informed about carrying out the research. Next, the directors and coordinators of the YEE who accepted to be part of the study gave their consent via a signed agreement. This study was implemented in the weekly hours corresponding to the tutorial hours of each course. In the participating educational centers, the directors informed the parents and the legal tutors, all families provided their written informed consent before starting this investigation. Once the participation of the educational centers was confirmed, the researchers agreed with the directors a specific date to visit the center and hand out the scale. The data collection in the educational centers was done between May 15th and July 15th 2018. The research team presented the fulfillment regulations and counseled the participants when they had doubts about filling out the scale. The aim of the investigation was explained to the students and anonymous participation in the data treatment was obtained. The tutors were present at all times. The scale was presented online [82] and was filled out in the computer classrooms in a 60-minute session.

### Ethical declaration

This investigation was approved and reviewed, including ethical aspects, by committee of the Ministry of Economy and Competitiveness (Spain, EDU2013-42936-P).

## Results

### Qualitative results

#### 1) Theoretical foundations and generation of items

**Fig 1:**
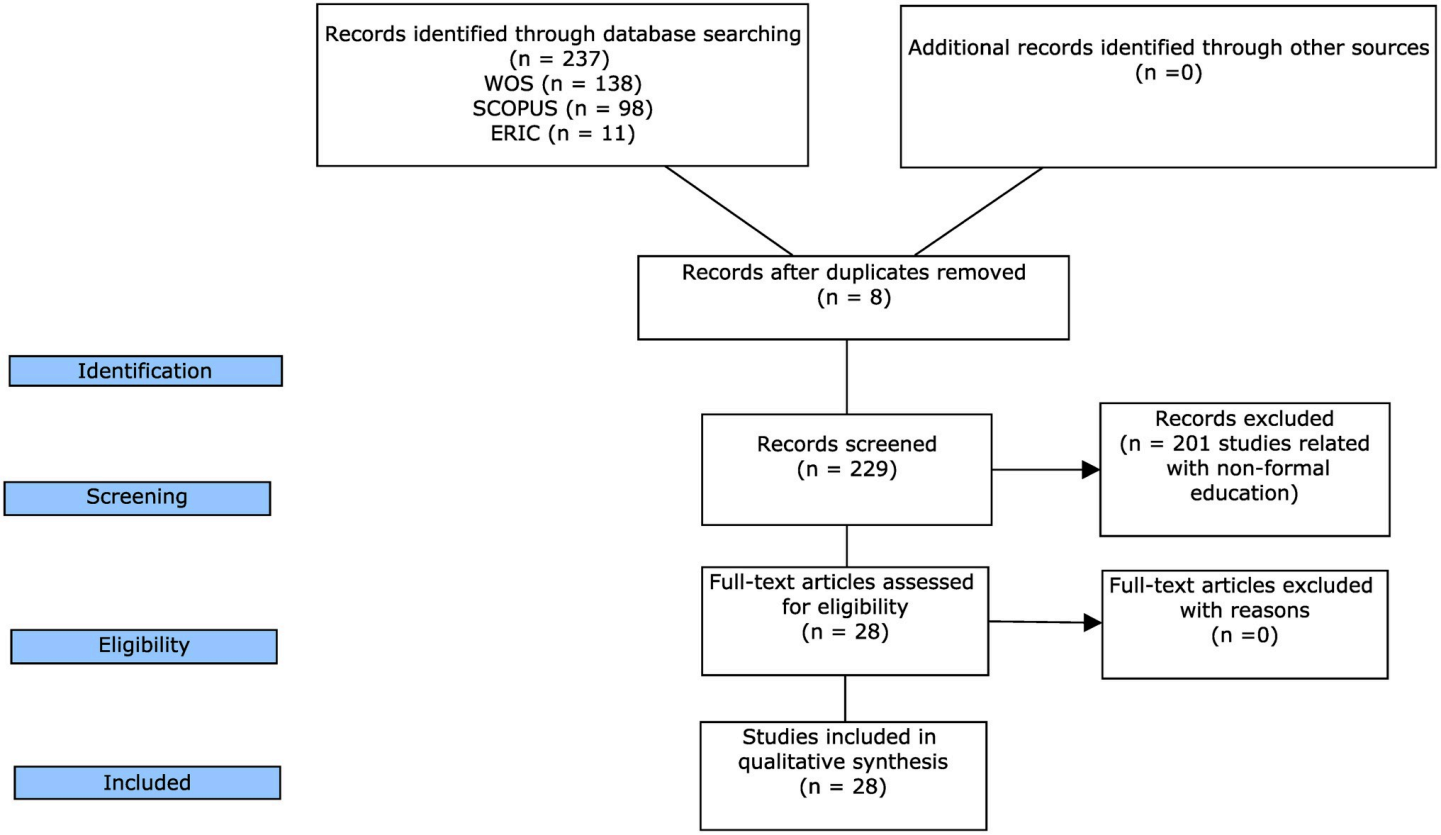
PRISMA flowchart. Modified from Moher, et al. (2009) [44].

237 research works were identified in the bibliographic search. 8 articles were excluded due to duplications and 201 articles were eliminated, as they were studies related with non-formal education (an exclusion criterion). Hence, on the expert judgment 28 studies were evaluated (Fig 1).

The degree of agreement concerning the content analysis among the experts in the Fleiss’ Kappa index [47] was K = .79, considered as acceptable [83, 84]. Basing ourselves on the literature reviewed about the conceptualization, composition and evaluation of entrepreneurship competency, he progressive model of entrepreneurship education in the EU countries [85], and the levels defined in the “EntreComp Proggresion Model” of the European Framework for Entrepreneurial Competence [86], a conceptual base was created to do the qualitative study with detailed interviews to expert teachers (8) and students (12). The systematic review of the bibliography, along with the data extracted from the detailed interviews with both groups of participants, enabled the research team to, on the one hand, configure the Basic Scale of Entrepreneurial Competencies in three dimensions: Operations and Marketing Competencies (OMC), Competencies in Socio-Business and Legal Organization (CSBLO) and Economic-Financial Competencies (EFC). On the other hand, 56 items were generated, eliminating 12 for redundancy. Thus, 44 items remained in a 4-point Likert scale: 1. Not at all; 2. Little; 3. Enough; and 4. A lot.

#### 2) Content validity

The data facilitated by the experts were compiled in the qualitative assessment of the formal and written aspects of the items. According to the review criterion, the choice of items to be revised by at least one expert, the writing of 17 items of the 44 items evaluated was modified. In the CVR, and basing ourselves on the criterion of Lawshe, 23 items which obtained scores lower than.62 were eliminated. In the CVI analysis 7 items were excluded as they did not reach values ≥.80. A total of 14 items were thus left. The CVR of the scale was.89 and the score of the CVI was.92, revealing that the Basic Scale of Entrepreneurial Competencies showed a high consistency in the content validity.

#### 3) Face validity

No relevant change was introduced in this sub-phase, as in the application of formal and conceptual problems categories no item was observed that produced a serious difficulty of conceptual or formal comprehensibility. Generally speaking, the students of the focus group via Think-aloud protocols described that the items were legible and comprehensible.

### Quantitative results

#### 1)Descriptive analyses and discrimination indices of the BCES

Table 3 shows the descriptive analyses and the discrimination indices of the items. The answers to the set of items show average scores of between 1.77 and 2.29, and standard deviations above one within a response range which goes from 1 to 3 [87]. The asymmetry and kurtosis indices show that the 14 items have values within the range ±1 [88]. The corrected item-total correlation attained scores >.45, showing a good degree of homogeneity(Table 3).

**Table 3:** Item descriptive statistics and discrimination indices.

Items No.	Descriptions of the items	M	SD	Skew.	Kurt.	I-tcd.
1	To create a firm’s brand advertising.	2.27	1.22	0.27	-1.44	0.75
2	To sell products and/or services.	2.22	1.18	0.35	-1.41	0.58
3	To design a product/service for a firm.	2.11	1.17	0.63	-1.19	0.63
4	To plan and organize the manufacturing of products/services in a firm.	1.93	1.11	0.76	-0.89	0.57
5	To appropriately set the prices of a product/service.	2.01	1.14	0.63	-1.11	0.60
6	To analyze the characteristics of a product/service.	1.99	1.09	0.62	-1.04	0.59
7	To analyze and identify what the customers of the products/services value.	2.29	1.16	0.22	-1.43	0.77
8	To organize people according to the work that they are going to do.	1.92	1.09	0.73	-0.93	0.55
9	To choose the most appropriate management model for a firm.	1.83	1.10	0.93	-0.65	0.50
10	To design a firm’s organigram.	1.85	1.06	0.91	-0.57	0.52
11	To set up a firm and to carry out the procedures to practice the activity.	1.77	1.07	1.04	-0.40	0.49
12	To elaborate a firm’s accounting book.	1.91	1.12	0.79	-0.86	0.56
13	To do a results account.	2.26	1.24	0.33	-1.36	0.67
14	To calculate a firm’s costs, profits and revenues.	2.25	1.19	0.47	-1.28	0.49

Notes. Mean = M. Standard Deviation = SD. Skewness = Skew. Kurtosis = Kurt. Item-total correlations dimension = I-tcd.

#### 2) Construct validity

*Exploratory Factor Analysis (EFA)*. The sample appropriateness was previously checked, applying the EFA to sample 1 (720). The KMO value is 0.96 and the Bartlett Sphericity test is chi-squared = 9042.7; p <.0001, showing that the matrix is suitable for the analysis. The solution extracted from the EFA was made up of three factors, with a total of 13 items, item 7 (Operations and Marketing Competencies) being removed for having a factor loading below.40 (Table 4): 1) Operations and Marketing Competencies (OMC). 2) Competencies in Socio-Business and Legal Organization (CSBLO). 3) Economic-Financial Competencies (EFC). The scale’s adjustment level is within the values considered appropriate (CFI = .99, GFI = .99, TLI = .96, RMSEA = .03 and SRMSR = .01). The three factors explain the 57.1% of the total variance of the scores. Particularly, dimension one, OMC, had 49.7% with 6 items. Dimension two, CSBLO, had 4.2% made up of 4 items. Dimension three, EFC, had 3.2% formed by 3 items. The description of the EFA-related data is in (Table 5).

**Table 4:** Dimensions of basic entrepreneurial competencies.

Dimensions	Nº of Items
Operations and Marketing Competencies (OMC)	7 items and 1 eliminated = 6 items
Competencies in Socio-Business and Legal Organization (CSBLO)	4 items
Economic-Financial Competencies (EFC)	3 items
3 dimensions	13 items

**Table 5:** Resulted obtained from EFA (sample of 720).

Items	Description of the factors and items	Factor Loadings			h^2^
	**F1. Operations and Marketing Competencies (OMC)**	1	2	3	
OMC 1	To create a firm’s brand advertising.	.671			.677
OMC 2	To sell products and/or services.	.587			.708
OMC 3	To design a product/service for a firm.	.560			.725
OMC 4	To plan and organize the manufacturing of products/services in a firm.	.544			.750
OMC 5	To appropriately set the prices of a product/service.	.530			.735
OMC 6	To analyze the characteristics of a product/service.	.475			.723
OMC 7	To analyze and identify what the customers of the products/services value.	.362			.731
	**F2. Competencies in Socio-Business and Legal Organization (CSBLO)**				
CSBLO 8	To organize people according to the work that they are going to do.		.653		.732
CSBLO 9	To choose the most appropriate management model for a firm.		.622		.608
CSBLO 10	To design a firm’s organigram.		.563		.617
CSBLO 11	To set up a firm and to carry out the procedures to practice the activity.		.430		.697
	**F3. Economic-Financial Competencies (EFC)**				
EFC 12	To elaborate a firm’s accounting book.			.852	.696
EFC 13	To do a results account.			.548	.576
EFC 14	To calculate a firm’s costs, profits and revenues.			.412	.674
**Eigenvalue**		24.75	1.65	1.61	-
**Explained Variance (%)**		49.7	4.2	3.2	-
**Cumulative Variance (%)**		49.7	53,9	57,1	-

Notes. h^2^ Communalities.

**Fig 2:**
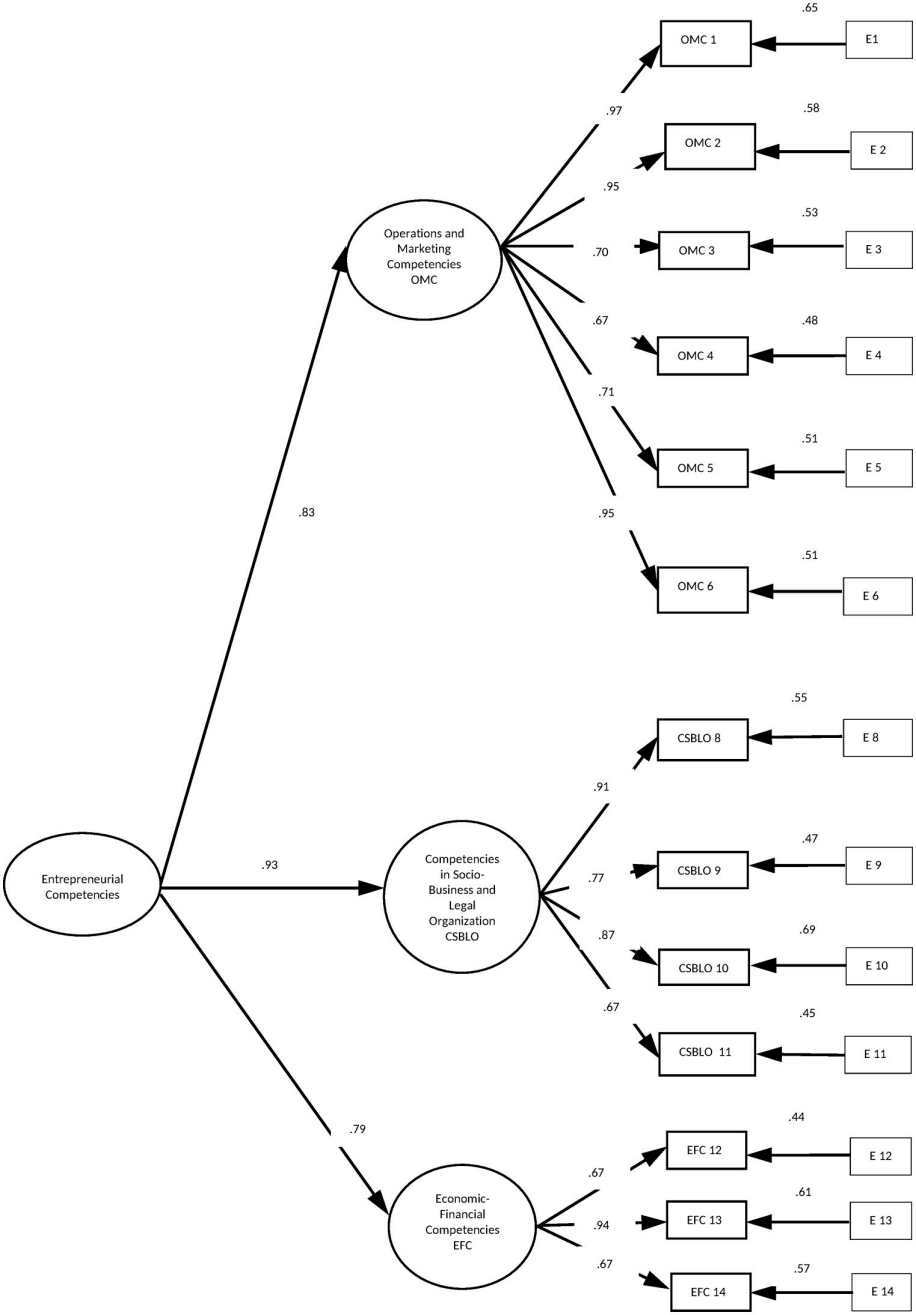
Metric model of the BSEC by CFA. Sample of 720 and standardized solution, p <.0001.

*Confirmatory Factor Analysis (CFA)*. To contrast the structure of the model extracted from the EFA, a second-order CFA was applied to sample 2 (720). The factorial weights were significant (p <.001), and both the absolute fit indices (GFI = .96, TLI = .97, RMSEA = .045 and the SRMSR = .07) and the incremental fit indices (CFI = .97, TLI = .97) showed that the model resulting from the EFA has a reasonably good fit with the hypothesized model [88] (Fig 2).

#### 3) Reliability

The Cronbach´s *α* coefficients of the three subscales have ranged between.79 and.90. The Cronbach´s *α* for the whole scale reached a value of.82. These results are interpreted as good [87]). These values, along with those found in the composite reliability coefficient (CR) and in the test-retest coefficient (ICC) for each of the subscales and for the total of the scale, show an appropriate reliability of the BCES scale [70, 90]. Table 6 shows a synthesis of these coefficients.

**Table 6:** Reliability and validity for the BSEC and its subescales.

Subescales	Items	CR	AVE	Cronbach´s *α* n = 720	ICC n = 50
Operations and Marketing Competencies (OMC)	1, 2, 3, 4, 5, 6	.92	.63	.90	.92
Competencies in Socio-Business and Legal Organization (CSBLO)	8, 9, 10, 11	.87	.70	.79	.82
Economic-Financial Competencies (EFC)	12, 13, 14	.87	.70	.79	.80
Basic Scale of Entrepreneurial Competencies (total)	13			.82	.92

Notes. Construct Reliability = CR. Average Variance Extracted = AVE. Cronbach´s *α* = Cronbach´s Alpha. Intraclass Correlation Coefficient = ICC.

#### 4) External validity

The items have shown good factorial weights ranging between the lowest weight of.412 and the highest of.852. The results of the evaluation of the convergent validity via the AVE with scores >.63 and of the CR with scores >.87, respectively, show that the three subscales fulfill the criterion >.40, thus obtaining appropriate properties of convergent validity [91]. Using the Fornell-Larcker criterion concerning the variance extracted, all the data indicates that the scores of each factor are greater than the square root of the correlations with the rest of the latent variables. So, the data show evidence of discriminant validity [91] (Table 7).

**Table 7:** Discriminant validity.

Subescales	Factor 1	Factor 2	Factor 3
Operations and Marketing Competencies	.*798*		
Competencies in Socio-Business and Legal Organization	.675	.*840*	
Economic-Financial Competencies	.764	.652	.*841*

Notes. Square root of the AVE in italics in the diagonal.

## Discussion

Effective entrepreneurial practices are globally considered a fundamental pillar for economic development and, likewise, these depend on an appropriate training of human capital [3, 93]. However, it is often believed that students automatically learn and accumulate entrepreneurial competence as an inevitable result of the set of pedagogical influences received. It cannot be denied that the cultural impact on the complete training of people has a positive relation with entrepreneurial training, but the challenges posed in current societies require people who can tackle the global transformation which occurs in the business world and labor market, hence specific educational attention is required [94]. The social need for entrepreneurial human capital training manifests itself strongly in the university and professional training levels and not so much in the basic teaching levels [95]. But the shaping of an entrepreneurial identity is a complex process which can be initiated from childhood and perhaps be a precursor of the sustainable entrepreneurial behaviors that systematically emerge in youth and adulthood [96]. Jayawama, Jones and Macpherson [97] have insisted on the idea of considering entrepreneurial human capital to be a consequence of a process of training developed from childhood. The relation between human capital and entrepreneurship involves, as numerous recent studies reveal [5], training in specific cognitive aspects and certain attitudes. The ethical and moral training of the entrepreneurial identity being of major relevance [98]. Attaining a high business development is not contradictory with ensuring an economic system that is sustainable and respectful with human dignity [99]. Ethical education in entrepreneurial education programs is essential for this goal. In this way, the basic levels of education acquire a notable relevance and the training of entrepreneurial competence also becomes a challenge for these first levels of the educational system and, maybe, particularly in secondary education, due to its propaedeutic character for higher studies and for its preparation for incorporation into the world of work. The accumulation of human capital is decisive for economic and social progress. Along with work experience and the influences of the family environment, the training which can be acquired in the school institution is fundamental. Among the aspects which make up the training of entrepreneurial human capital, acquired entrepreneurial competences, as well as the relevant attitudes and knowledge, are a determinant factor. The psychometric properties of the Basic Scale of Entrepreneurial Competencies for secondary education students are evaluated in this research. The European Union, in the framework of guidelines to their Member States for fostering the knowledge economy, points out the need to incorporate and develop business education in the lower levels of the educational system. Entrepreneurial competency is a training domain of entrepreneurship education, so attaining appropriate levels of development in entrepreneurial competency is one of the main aims of business education [100]. However, the assessment of entrepreneurial competency in adolescence or in earlier ages has been hardly studied. Specifically, the RediE study [62] and the Eurydice report [101] indicate the lack of rigorous assessment instruments related with business education in the compulsory education stages. In this sense, the Basic Scale of Entrepreneurial Competencies was developed to satisfy the need for an instrument which measures entrepreneurship competency in Secondary Education. This study has been implemented via mixed research procedures [102]. At the end of Phase 1 there were 14 items for the study of validity and reliability. In the EFA 1 item was eliminated because it had a factor loading less than.40 -(OMC 7) “To analyze and identify what the customers of the products/services value” -and 13 items with satisfactory factor loadings were kept. As a factor solution three factors were extracted from those 13 items. These items had satisfactory factor loadings in each corresponding factor. Firstly, a first-order factor structure made up of three factors was confirmed in the CFA and, later, a more parsimonious second-order factor solution was attained by extracting a sole “Basic Entrepreneurial Competency”. The absolute and incremental fit indices obtained appropriate scores, confirming the BSEC’ structural model. In this way, the results obtained in the content, construct and reliability validity establish a mono-factor model of entrepreneurship competency in the Secondary Education stage made up of the Basic Entrepreneurial Competency factor and shaped by three dimensions: Operations and Marketing Competencies, Competencies in Socio-Business and Legal Organization and Economic-Financial Competencies. These dimensions are congruent with the foundations of the entrepreneurial competencies of other previous studies [103–105]. However, the proportionality of competences described in these frameworks of competence references is greater than that shown in our scale. This factorial composition is in agreement with the initial levels of training of entrepreneurial competence described in the “EntreComp Proggresion Model” of the European Framework for Entrepreneurial Competence [86], and with the basic business education of the educational stage studied [106]. The psychometric properties indicate that the Basic Scale of Entrepreneurial Competencies is consistent and robust, allowing the evaluation of the degree of entrepreneurial competencies in the students. Among other features, its use serves to evaluate and detect the students’ training needs or find out the impact of business education programs on entrepreneurial competencies. This research on competencies, along with the previous research of basic business knowledge [42], enables an unprecedented research line in entrepreneurial education in this education stage. It is not only related with the construction of measurement scales which cover the existing gaps concerning reliable and valid scales, but also, especially, with building an evaluation model concerning the entrepreneurial qualification acquired by the students through the business knowledge and skills developed in entrepreneurial education programs. Specifically, in the future, it would consist of configuring, analyzing and evaluating a hypothetical model of entrepreneurial qualification with a tertiary hierarchical structure which combines knowledge and skills within the domain of entrepreneurial qualification. Ultimately, this approach would mean an original conceptual and metric framework concerning the implementation and evaluation of entrepreneurial education programs in which the BSEC and the BBKS [42]) would be additional configuration elements.

All the data indicate that the Basic Scale of Entrepreneurial Competencies is consistent and robust, enabling assessing the degree of development of entrepreneurial competencies in students with a sole instrument. This instrument contributes to reducing the shortcomings of valid and reliable instruments concerning the assessment of business education in this educational stage. Among others, its use will serve to assess and detect students’ training needs and to find out the effect of business education programs on entrepreneurial competencies [107]. The psychometric properties of the BSEC have been demonstrated, but this study also has some limitations: 1) it is the first time that the validation of the scale has been developed and it is not a universal instrument, more studies would be necessary in other school and age contexts, business education programs, types of schools and educational stages (Primary Education, High School Degree or Professional Training); 2) as the sex has not been analyzed in the study of the reliability and validity, research in this sense ought to be carried out; 3) nor have the socio-demographic and economic variables been studied and it will be necessary to introduce this type of variables in future researches.

## Conclusion

The results of the study indicate that the Basic Scale of Entrepreneurial Competencies for Secondary Education has good psychometric properties. Centered on entrepreneurial competency, this Scale aims to offset the current lack of validated instruments in the basic levels of formal education (S3 and S4 Files). It is also useful for future researches related with the training of entrepreneurial competencies within the school system, as well as for the design and development of innovative entrepreneurial practices which mean to explore new forms of business education, including a fundamental range of business activities.

## Supporting information

S1 Checklist PRISMA checklist. (PDF)

S1 File Interview guides in English version. (DOC)

S2 File Interview guides in Spanish version. (DOC)

S3 File Basic Scale Entrepreneurial Competencies for Secondary Education Students (BSEC)- Spanish version. (DOC)

S4 File Basic Scale Entrepreneurial Competencies for Secondary Education Students (BSEC)- English version. (DOC)

## References

[1] Stiglitz JE, Greenwald BC. Creating a Learning Society: A New Approach to Growth, Development and Social Progress. New York: Columbia University Press; 2014.

[2] Barnard A, Pittz T, Vanevenhoven J. Entrepreneurship education in U.S. community colleges: A review and analysis. J. Small Bus. Enterp. Dev. 2019; 26(2): 2–208. 10.1108/JSBED-06-2018-0178

[3] Gachino CG, Worku GB. Learning in higher education: Towards knowledge, skills and competency acquisition. Int. J. Educ. Manag. 2019; 33(7): 7–1770. 10.1108/IJEM-10-2018-0303

[4] Martin BC, McNally JJ, Kay MJ. Examining the formation of human capital in entrepreneurship: A meta-analysis of entrepreneurship education outcomes. J Bus Ventur. 2013; 28(2): 2–224. 10.1016/j.jbusvent.2012.03.002

[5] Unger JM, Rauch A, Frese M, Rosenbusch N. Human capital and entrepreneurial success: A meta-analytical review. J. Bus. Ventur. 2011; 26(3): 3–358. 10.1016/j.jbusvent.2009.09.004

[6] Order ECD/ 65/2015, of January 9, describing the relationships between the competences, content and evaluation criteria of primary education, compulsory secondary education and high school. (BOE, Official State Bulletin, number 25, of 29-01-2015).

[7] Mitchelmore S, Rowley J. Entrepreneurial competencies: a literature review and development agenda. Int. J. Entrepreneurial Behav. Res. 2010; 16(2): 2–111. 10.1108/13552551011026995

[8] Mehta A, Yoon E, Kulkarni N, Finch D. An exploratory study of entrepreneurship education in multidisciplinary and multicultural environment. Res. Econ. 2000; 54(1): 1–56. 10.1006/reec.1999.0225

[9] Menke C. Unravelling entrepreneurial competencies and their relation to entrepreneurial intent. J. Bus. Ventur. 2018; 10(6): 6–687. 10.1504/IJEV.2018.095304

[10] Rippa P, Ponsiglione C, Bocanet A, Capaldo G, Zollo G. Do new ventures explore, exploit of both? A case-based analysis of six innovative Italian sturp-ups. Int. J. Entrepreneurial Behav. Res. 2019; 25(7): 7–1536. 10.1108/IJEBR-12-2018-0817

[11] Al Mamun A, Fazal SA. Effect of entrepreneurial orientation on competences and micro-enterprise performance. APJIE. 2018; 12(3): 3–358. 10.1108/APJIE-05-2018-0033

[12] Kadam R, Rao S, Abdul WK, Jabeen SS. Impact of cultural intelligence on SME performance. The mediating effect of entrepreneurial orientation. JOEPP. 2019; 6(3): 3–185. 10.1108/JOEPP-12-2018-0101

[13] Wang SM, Yueh HP, Wen PC. How the New Type of Entrepreneurship Education Complements the Traditional One in Developing Entrepreneurial Competencies and Intention. Front. Psychol. 2019; 10: 2048. 10.3389/fpsyg.2019.02048 31572260 PMC6753869

[14] Fey THC, Ahmad NH, Ramayah T. Entrepeneur education: does prior experience matter? J. Eur. Ind. Train. 2012; 15: 65–82.

[15] Vanevenhoven J, Liguori EW. The impact of entrepreneurship education: introducing the entrepreneurship education project. J. Small Bus. Strategy. 2013; 51(3): 3–328. 10.1111/jsbm.12026

[16] Patel PC, Ganzach Y. Returns to balance in cognitive skills for the self-employed: evidence from 18 countries. Small. Bus. Econ. 2019; 52(1): 1–109. 10.1007/s11187-018-0018-4

[17] Samad NA, Ahmad WMRW, Sern LC, Harun H, Awang H, Noor SNFM. Exploring domains and elements for behavioral competency and employability skills. JTET. 2018; 10(1): 1–90.

[18] Buzady Z, Almeida F. FLIGBY-A Serious Game Tool to Enhance Motivation and Competencies in Entrepreneurship. Informatics. 2019; 6(27): 27–19. 10.3390/informatics6030027

[19] Kassean H, Vanevenhoven J, Liguori E, Winkel DE. Entrepreneurship education: A need for reflection real-world experience and action. Int. J. Entrepreneurial Behav. Res. 2015; 21(5): 5–708. 10.1108/IJEBR-07-2014-0123

[20] Dickson PH, Solomon GT, Weaver KM. Entrepeneurial selection and success: does education matter? J. Small. Bus. Enterp. Dev. 2008; 15(2): 2–258. 10.1108/14626000810871655

[21] Machi L, McEvoy B. The literature review: Six steps to success. Thousand Oaks, CA: Corwin Press; 2016.

[22] Pittaway L, Cope J. Entrepreneurship education: A systematic review of the evidence. ISBJ. 2007; 25(5): 5–510.

[23] Lackeus M, Savetun C. Assessing the Impact of Enterprise Education in Three Leading Swedish Compulsory Schools. J. Small Bus. Manag. 2019; 57 (Special Issue, Supplement 1): 33–59. 10.1111/jsbm.12497

[24] Dinning T. Articulating entrepreneurial competencies in the undergraduate curricular. Educ. Train. 2019; 61(4): 4–444. 10.1108/ET-09-2018-0197

[25] Choi M, Park E. The effects of youth education on entrepreneurship. International Information Institute (Tokyo). 2015; 18(5): 5–1990.

[26] Liñán F, Ceresia F, Bernal A. Who intends to enroll in intrepreneurship education? Entrepreneurial self-identity as a precursor. Entrepreneurship Education and Pedagogy. 2018; 1(3): 3–242. 10.1177/2515127418780491

[27] Fayolle A, Gailly B. From craft to science: Teaching models and learning processes in entrepreneurship education. J. Eur. Ind. Train. 2008; 32(7): 7–593. 10.1108/03090590810899838

[28] Fayolle A. (editor) A Research Agenda for Entrepreneurship Education. Cheltenham, UK, Northampton, MA, USA: Edward Elgar Publishing; 2018.

[29] Chell E. Review of skill and the entrepreneurial process. Int. J. Entrepreneurial Behav. Res. 2013; 19(1): 1–31. 10.1108/13552551311299233

[30] Brophy M, Kiely T. Competencies: A new sector. J. Eur. Ind. Train. 2002; 26(2/3/4): 165–276. 10.1108/03090590210422049

[31] Vargas-Halabi T, Mora-Esquivel R, Siles B. Intrapreneurial competencies: development and validation of a measurement scale. EJM BE. 2017; 26(1): 1–111. 10.1108/EJMBE-07-2017-006

[32] Tolentino LR, Sedoglavich V, Lu VN, García PRJM, Restubog SLD. The role of career adaptability in predicting entrepreneurial intentions: A moderated mediation model. J. Vocat. Educ. Train. 2014; 85(3): 3–412. doi: 10-1016/j.jub.2014.09.002

[33] Nab J, Oost H, Pilot A, van Keulen H. Measurement of the Ability of Science Students to Recognize Business Opportunities. In: Marriot N, editor. European Conference on Entrepreneurship and Innovation. Winchester, UK: Winchester University; 2008. pp. 189–196.

[34] Hazudin SF, Kader M, Tarmuji NH, Ishak M. Ali R. Discovering Small Business Start up Motives, Success Factors and Barriers: A Gender Analysis. In: Sanusi ZM, Zakaria NB, Othman AA, Musirin I, Dahlan JM, editors. BookSeries: Procedia Economics and Finance. 31, 436-443. doi: 10-1016/S2212-5671(15)01218-6

[35] Chandler GN, Jansen E. The founder’s self-assessed competence and venture performance. J. Bus. Ventur. 1992; 7(3): 3–36. 10.1016/0883-9026(92)90028-P

[36] Bird B. Towards a theory of entrepreneurial competency. In: Katz J, Corbet AC, editors. Seminal Ideas for the Next Twenty-Five Years of Advances (Advances in Entrepreneurship, Firm Emergence and Growth, Vol. 21). Bingley, UK: Emerald Publishing Limited; 2019. pp.115–131.

[37] Van der Heijden B.I.J.M. The development and psychometric evaluation of a multidimensional instrument of professional expertise. High Abil. Stud. 2000; 11: 9–79. 10.1080/713669175

[38] Man T, Lau T. Entrepreneurial competencies of SME owner/managers in the Hong Kong services sector: a qualitative analysis. J. Enterprising Cult. 2000; 8(3): 3–54. 10.1142/S0218495800000139

[39] Markman GD, Balkin DB, Baron RA. Inventors and new venture formation: the effects of general self-efficacy and regretful thinking. Entrep. Theory Pract. 2002; 27(2): 2–65. 10.1111/1540-8520.00009

[40] Smith B, Morse E. Entrepreneurial Competencies: Literature Review and Best Practices, Small Business Policy Branch, Industry Canada. Ottawa: ON; 2005.

[41] Kyndt E, Baert H. Entrepreneurial competencies: Assessment and predictive value for entrepreneurship. J. Vocat. Behav. 2015; 90: 13–25. 10.1016/j.jvb.2015.07.002

[42] Bernal-Guerrero A, Cárdenas-Gutiérrez AR, Montoro-Fernández E. Basic business knowledge scale for secondary education students. Development and validation with Spanish teenagers. PLoS One. 2020; 15(7), 713–731. 10.1371/journal.pone.0235681 32634821 PMC7340510

[43] Creswell JW, Plano Clark VL. Designing and conducting mixed methods research. 3rd ed. Thousand Oaks, CA: Sage; 2018.

[44] Moher D, Liberati A, Tetzlaff J, Altman DG, The PRISMA Group. Preferred Reporting Items for Systematic Reviews and Meta-Analyses:The PRISMA Statement. PLoSMed. 2009; 6(7): 1–6. 10.1371/journal.pmed.1000097 PMC3090117 21603045

[45] Moher D, Shamseer L, Clarke M, et al. Preferred reporting items for systematic review and meta-analysis protocols (PRISMA-P) 2015 statement. Syst Rev. 2015; 4(1): 1–9. 10.1186/2046-4053-4-1 25554246 PMC4320440

[46] Braun V, Clarke V. Using thematic analysis in psychology. Qual. Res. Psychol. 2006; 3(2): 2–101. 10.1191/1478088706qp063oa

[47] Fleiss JL, Cohen J, Everitt BS. Large sample standard errors of kappa and weighted kappa. Psychol Bull. 1969; 72(5): 5–327. 10.1037/h0028106

[48] Hallgren KA. Computing Inter-Rater Reliability for Observational Data. An Overview and Tutorial. Tutor Quant Methods Psychol. 2012; 8(1), 23–34. 10.20982/tqmp.08.1.p023 22833776 PMC3402032

[49] Escobar-Pérez J, Cuervo-Martínez A. Validez de contenido y juicio de expertos: Una aproximación a su utilización. Avances en Medición. 2008; 6: 27–36.

[50] Lannoy A. L’utilisation du judgement d’experts en sûreté de fonctionnement. Paris: Tec & Doc Lavoisier; 2001.

[51] Ayre C, Scally AJ. Critical Values for Lawshe’s Content Validity Ratio: Revisiting the Original Methods of Calculation. Meas. Eval. Couns. Dev. 2014; 47(1): 1–86.

[52] Lawshe CH. A quantitative approach to content validity. Pers. Psychol. 1975; 28: 563–575. 10.1111/j.1744-6570.1975.tb01393.x

[53] Polit DF, Beck CT. The content validity index: Are you sure you know what’s being reported? Critique and recommendations. RINAH. 2006; 29: 489–497. 16977646 10.1002/nur.20147

[54] Polit DF, Beck CT, Owen SV. Is the CVI an Acceptable Indicator of Content Validity? Appraisal and Recommendations. RINAH. 2007; 30: 459–467. 17654487 10.1002/nur.20199

[55] Davis LL. Instrument review: Getting the most from a panel of experts. Appl Nurs Res. 1992; 5: 194–197. 10.1016/S0897-1897(05)80008-4

[56] Cowan J. The potential of cognitive think-aloud protocols for educational action-research. Act. Learn. High. Educ. 2019; 20(3): 3–232. 10.1177/1469787417735614

[57] Ericsson KA, Simon HA. Verbal Reports as data. Psychol. Rev. 1980; 87(3): 3–251. 10.1037/0033-295X.87.3.215

[58] Ericsson KA, Simon HA. Protocol analysis: Verbal reports as data. Cambridge, MA: The MIT Press; 1984.

[59] Gu P. To code or not to code: Dilemmas in analysing think-aloud protocols in learning strategies research. System. 2014; 43(1): 1–81. 10.1016/j.system.2013.12.011

[60] Charters E. The use of think-aloud methods in qualitative research an introduction to think-aloud methods. Brock Educ. J. 2003; 12(2): 2–82. 10.26522/brocked.v12i2.38

[61] European Commission. Entrepreneurship education in Europe: fostering entrepreneurial mindsets through education and learning. In: Final Proceedings of the Conference on Entrepreneurship Education in Oslo. 2006. [Cited 2020 October 14]. Available from: https://ec.europa.eu/growth/content/entrepreneurship-education-europe-fostering-entrepreneurial-mindsets-through-education-anden.

[62] Diego I, Vega JA. La educación para el emprendimiento en el sistema educativo español. Año 2015. Estudio RediE. Madrid: MEC; 2015.

[63] Izquierdo I, Olea J, Abad FC. Exploratory factor analysis in validation studies: Uses and recommendations. Psicothema. 2014; 23(1): 1–400. 10.7334/psicothema2013.349 25069561

[64] Worthington RL, Whittaker TA. Scale Development Research: Acontent Analysis and Recommendations for Best Practices. TCP. 2006; 6(34): 34–838. 10.1177/0011000006288127

[65] Nunnally JC, Bernstein IH. Psychometric Theory. 3rd ed. New York: McGraw-Hill; 1994.

[66] Flora DB, LaBrish C, Chalmers RP. Old and new ideas for data screening and assumption testing for exploratory and confirmatory factor analysis. Front Psycho. 2012; 3(55), 1–21. 10.3389/fpsyg.2012.00055 22403561 PMC3290828

[67] Harrington D. Confirmatory factor analysis. New York: McGraw-Hill; 1994.

[68] Courtney MGR, Gordon M. Determining the number of factors to retain in EFA: Using the SPSS R-Menu v2.0 to make more judicious estimations. Pract. Assess. Res. Evaluation. 2013; 18(8): 8–14. 10.2147/JHL.S35483

[69] Brown TA. Confirmatory factor analysis for applied research. 2nd ed. New York: Guilford Press; 2015.

[70] Hair JF, Anderson RE, Babin BJ, Black WC, Multivariate data analysis. 8nd ed. Hampshire: Cengage Learning EMEA; 2018.

[71] Hoyle R. Structural equation modeling. Concepts, issues, and applications. London: Sage; 1995.

[72] Valera J, Rial A, García E. Presentación de una escala de satisfacción con los servicios sanitarios de atención primaria. Psicothema. 2003; 15(4): 656–661.

[73] Hu LT, Bentler PM. Cutoff criteria for fit indexes in covariance structure analysis: Conventional criteria versus new alternatives. Struct Equ Model. 1999; 6(1): 1–55. 10.1080/10705519909540118

[74] Hulley SB, Cummings SR. Diseño de la investigación clínica un enfoque epidemiológico. Barcelona: Ediciones Doyma, S.A.; 1993.

[75] Bartko JJ. The intraclass correlation coefficient as a measure of reliability. Psychol Rep. 1966; 19: 3–11. 10.2466/pr0.1966.19.1.3 5942109

[76] Terry KK, Mae YL. A Guideline of Selecting and Reporting Intraclass Correlation Coefficients for Reliability Research. J Chiropr Med. 2016 6; 15(2): 2–163. 10.1016/j.jcm.2016.02.012 PMC4913118 27330520

[77] Fornell C, Larcker DF. Evaluating structural equation models with unobservable variables and measurement error. J Mark Res. 1981; 18(1): 1–50. 10.2307/3151312

[78] Lorenzo-Seva U, Ferrando PJ. Evaluating structural equation models with unobservable variables and measurement error. Behav Res Methods Instrum Comput. 2006; 38(1): 1–91. 10.3758/BF03192753

[79] Bentler PM. EQS 6 Structural Equations Program Manual. Encino, CA: Mulivariate Software, Inc; 2006.

[80] Chin WW. PLS-Graph. Version 3.00. build 1060. Texas: University of Houston; 2004.

[81] IBM. SPSS statistics for Windows. Version 21.0. Armonk, NY: IBM; 2012.

[82] Drasgow F, editor. Technology and testing. Nueva York: Routledge; 2016.

[83] Altman DG. Practical statistics for medical research. New York: Chapman and Hall; 1991.

[84] Landis JR, Koch GG. The measurement of observer agreement for categorical data. Biometrics. 1977; 33(1): 1–74. 10.2307/2529310 843571

[85] McCoshan A, Lloyd P, Blakemore M, Gluck D, Betts J, Lepropre M, et al. Towards Greater Cooperation and Coherence in Entrepreneurship Education. Report and Evaluation of the Pilot Action High Level Reflection Panels on Entrepreneurship Education initiated by DG Enterprise and Industry and DG Education and Culture. 2010. [cited 2020 November 14]. Available from: http://ec.europa.eu/enterprise/policies/sme/promotingentrepreneurship/educationtrainingentrepreneurship/reflectionpanels/files/entreducatinpanelen.pdf.

[86] Bacigalupo M, Kampylis P, Punie Y, van den Brande G. EntreComp: The Entrepreneurship Competence Framework. Luxembourg: Publication Office of the European Union; 2016.

[87] Dunn TJ, Baguley T, Brunsden V. From alpha to omega: a practical solution to the pervasive problem of internal consistency estimation. Br J Psychol. 2014; 105(3), 399–412. 10.1111/bjop.12046 24844115

[88] Muñiz J. Introducción a la Psicometría. Teoría clásica y TRI. Madrid: Pirámide; 2018.

[89] Schreiber JB, Nora A, Stage FK, Barlow EA, King J. Reporting structural equation modeling and confirmatory factor analysis results: A review. J Educ Res. 2006; 99(6): 6–338. 10.3200/JOER.99.6.323-338

[90] Kline RB. Principles and practice of structural equation modeling. 4th ed. New York and London: The Guilford Press; 2016.

[91] Nunnally J. Psychometric methods. New York: McGraw-Hill; 1978.

[92] Hair J, Hult G, Ringle C, Sarstedt M, Gudergan S. Advanced issues in Partial least Squares Structural Equation Modeling. Los Angeles: Sage; 2017.

[93] Hanushek E, Woessmann L. The role of cognitive skills in economic development. J. Econ. Lit. 2008; 46(3): 3–668. 10.1257/jel.46.3.607

[94] Bernal A. Competencia emprendedora e identidad personal. Una investigación exploratoria con estudiantes de Educación Secundaria Obligatoria. Rev. de Educ. 2014; 363: 384–411. 10.4438/1988-592X-Re-2012-363-192

[95] Martin BC, McNally JJ, Kay MJ. Examining the formation of human capital in entrepreneurship: A meta-analysis of entrepreneurship education outcomes. J. Bus. Ventur. 2013; 28(2): 2–224. 10.1016/j.jbusvent.2012.03.002

[96] Fayolle A, Gailly B. The impact of entrepreneurship education on entrepreneurial attitudes and intention: Hysteresis and persistence. J. Small Bus. Manag. 2015; 53(1): 1–93. 10.1111/jsbm.12065

[97] Jayawama D, Jones O, Macpherson A. Becoming an entrepreneur: the unexplored role of childhood and adolescent human capital. In: Rae D, Wang C.L, editors. Entrepeneurial Learning: New Perspectives in Research, Education and Practice. London-New York: Routledge; 2015. pp. 45–72

[98] Mendez MJ, Vollrath DA, Ritter L. I and We: Does Identity Explain Undergraduates´ Ethical Intentions? J. Bus. Ethics Educ. 2018; 15: 75–98. 10.5840/jbee2018155

[99] Jones P, Hillier D, Comfort D. The sustainable development goals and business. IJSRM. 2016; 5(2): 2–48.

[100] Lackéus M. Entrepreneurship in education–What, why, when, how. Paris: OECD. [cited 2020 April 2]. Available from: https://www.oecd.org/cfe/leed/BGP_Entrepreneurship-in-Education.pdf

[101] European Commission/EACEA/Eurydice Entrepreneurship Education at School in Europe. Eurydice Report. Luxembourg: Publications Office of the European Union; 2016.

[102] Onwuegbuzie AJ, Bustamante RM, Nelson JA. Mixed research as a tool for developing quantitative instruments. J. Mix. Methods Res. 2010; 4(1): 56–78. 10.1177/1558689809355805

[103] Mitchelmore S, Rowley J. Entrepreneurial competencies: A literature and development agenda. International. Int. J. Entrepreneurial Behav. Res. 2010; 16(2): 2–111. 10.1108/13552551011026995

[104] Palacios-Marqués D, Guijarro M, Martí M, Alguacil MP. Social entrepreneurship and organizational performance: A study of the mediating role of distinctive competencies in marketing. J. Bus. Res. 2019; 101: 426–432. 10.1016/j.jbusres.2019.02.004

[105] Davies P, Syed F, Appleyard L. Secondary School Students’ Understanding of the Financial System In: Wuttke E, Seifried J, Schumann S, editors. Economic Competence and Financial Literacy of Young Adults: Status and Challenges. Opladen-Berlin-Toronto: Verlag Barbara Budrich; 2016. pp. 45-72.

[106] Organic Law 8/2013, of December 9, for the improvement of quality educational. (BOE, Official State Bulletin, number 295, of 10-12-2013).

[107] Sánchez JC. The Impact of an Entrepreneurship Education Program on Entrepreneurial Competencies and Intention. J. Small Bus. Manag. 2013; 51(3): 3–465. 10.1111/jsbm.12025

