# Supplementary material for: Construction and validation of the Basic Scale of Entrepreneurial Competencies for the Secondary Education level. A study conducted in Spain
Source: PLoS One. 2021 Apr 15;16(4):e0249903. doi: 10.1371/journal.pone.0249903 (PMC8049328; doi:10.1371/journal.pone.0249903)
Supplement: S1 File — (DOC) [file pone.0249903.s002.doc]

**INTERVIEW FOR EXPERTS IN ENTREPRENEURSHIP EDUCATION IN THE STAGES OF COMPULSORY EDUCATION**

**Interviewer's Topic Guide**

**Introduction to Interview**

First of all, we express our appreciation for your participation.

With this interview we try to know your opinion, as an expert teacher in entrepreneurship education, about entrepreneurial competencies concerning the programs of this type of education. Your valuable information will help us to design an assessment instrument for students´ basic entrepreneurial competencies, which is part of an extensive research on entrepreneurship education in the stages of compulsory education.

Comment about confidentiality:

This interview will be recorded and later transcribed, guaranteeing that all the data from the conversation will be confidential and anonymous. With your permission, we will begin recording.

**Start of the interview**

**Conceptualization of entrepreneurial competencies of entrepreneurship education programs**

1) Could you define what entrepreneurial competencies are?

2) Could you describe which entrepreneurial competencies you teach in entrepreneurship education programs?

3) Could you tell me the characteristics of these entrepreneurial competencies?

**Assessment of entrepreneurial competencies of entrepreneurship education programs**

From your perspective as a teacher:

4) Which are the most relevant entrepreneurial competencies to teach? Why?

5) Which are the least relevant entrepreneurial competencies to teach? Why?

6) Do you think the entrepreneurial competencies you teach are useful for the professional development of students? Why?

7) Apart from these competencies, could other types of entrepreneurial competencies be imparted?

7.1) If yes, why? and which ones?

7.2) If not, why can't another type of entrepreneurial competencies be imparted?

**Teaching experience and entrepreneurial competencies of entrepreneurship education programs**

From your experience as a teacher:

8) Would it be convenient to teach students all the entrepreneurial competencies about the design of a project/business plan?

If not, which ones would you teach? Why?

If yes, why?

9) If you did not have time to teach all the entrepreneurial competencies of the program, what content would you prioritize to teach your students? Why?

10) Thinking of compulsory secondary education as an educational stage of basic business training, what entrepreneurial competencies would you teach students for their subsequent incorporation into vocational training or pre-university studies?

11) What are the criteria you use to select and prioritize entrepreneurial competencies for students?

12) Thinking about your teaching experience and the business context, do you think that the program's entrepreneurial competencies are adapted to the training needs of companies? Why?

**Closure of the interview**

We have no further questions, although we would like to offer you the opportunity to add any comments or observations about the business knowledge that are being taught, if you wish.

We reiterate our gratitude for your participation.

**INTERVIEW FOR SECONDARY EDUCATION STUDENTS PARTICIPATING IN ENTREPRENEURSHIP EDUCATION PROGRAMS**

**Interviewer's Topic Guide**

**Introduction to Interview**

First of all, we express our appreciation for your participation.

With this interview we try to know your opinion, as an expert teacher in entrepreneurship education, about entrepreneurial competencies concerning the programs of this type of education. Your valuable information will help us to design an assessment instrument for students´ basic entrepreneurial competencies, which is part of an extensive research on entrepreneurship education in the stages of compulsory education.

Comment about confidentiality:

This interview will be recorded and later transcribed, guaranteeing that all the data from the conversation will be confidential and anonymous. There are no right or wrong answers. You are completely free to express your opinion and you can request any clarification you need during the interview. With your permission, we will begin recording.

**Start of the interview**

**Typology of entrepreneurial competencies in entrepreneurship education programs**

As a student:

1) What entrepreneurial competencies are learned in the entrepreneurship education program?

2) Which would be the entrepreneurial competencies needed to develop a business project or plan?

From your perspective:

3) Which are the most important entrepreneurial competencies you've learned? Why?

4) Which are the least important entrepreneurial competencies you've learned? Why?

You've participated in entrepreneurship education programs in previous courses, thinking about them:

5) What type of entrepreneurial competencies did you learn in those programs?

5.1) Are they linked to the competencies you are learning in this academic year?

5.2) In which way?

5.3) Could you give any examples of the link or relationship between competencies from previous years and the current ones?

5.4) Considering everything you have learned, how would you classify the acquired competencies?

**Characteristics of entrepreneurial competencies** **in entrepreneurship education programs**

From your perspective as a student:

6) What are entrepreneurial competencies? How would you define it?

7) In general, how are the entrepreneurial competencies?

More concretely:

7.1) Are they linked to real life?

7.2) Do you think that the entrepreneurial competencies learned are immediately applicable to the company? Why? Can you give any examples or describe which competencies you think is more applicable and which is not?

7.3) Do you want to highlight any more characteristics of these entrepreneurial competencies?

Comparing the entrepreneurial competencies of the entrepreneurship education program of this academic year with the entrepreneurship education programs of previous courses:

8) Which are the differences or similarities of these entrepreneurial competencies?

8.1) Could you describe some examples of the differences or similarities?

9) With the competencies gained, would you dare to start a business? Would any unacquired knowledge be required?

**Closure of the interview**

We have no further questions, although we would like to offer you the opportunity to add any comments or observations about the entrepreneurial competencies that are being taught, if you wish.

We reiterate our gratitude for your participation.
