# Supplementary material for: Construction and validation of the Basic Scale of Entrepreneurial Competencies for the Secondary Education level. A study conducted in Spain
Source: PLoS One. 2021 Apr 15;16(4):e0249903. doi: 10.1371/journal.pone.0249903 (PMC8049328; doi:10.1371/journal.pone.0249903)
Supplement: S2 File — (DOC) [file pone.0249903.s003.doc]

**ENTREVISTA PARA EXPERTOS EN EDUCACIÓN EMPRESARIAL EN LAS ETAPAS DE LA EDUCACIÓN OBLIGATORIA**

**Guía temática del entrevistador**

**Introducción a la entrevista**

En primer lugar, le expresamos nuestro agradecimiento por su participación.

Con esta entrevista pretendemos conocer su opinión, como docente experto en educación empresarial, sobre las habilidades empresariales que forman parte de los programas de este tipo de educación. Su valiosa información nos será útil para diseñar un instrumento de evaluación sobre habilidades empresariales básicas del alumnado, que forma parte de una amplia investigación sobre la educación empresarial en las etapas de la educación obligatoria.

Comentario sobre la confidencialidad:

Esta entrevista será grabada y posteriormente transcrita, garantizándole que todos los datos de la conversación serán confidenciales y anónimos. Con su permiso, vamos a comenzar a grabar.

**Inicio de la entrevista**

**Conceptualización de las habilidades empresariales de los programas de educación emprendedora**

1) ¿Podría definirme qué son las habilidades empresariales?

2) ¿Me puede describir cuáles son las habilidades empresariales que enseña en los programas de educación emprendedora?

3) ¿Me podría decir las características de esas las habilidades empresariales?

**Valoración de las habilidades empresariales de los programas de educación emprendedora**

Desde su punto de vista como docente:

4) ¿Cuáles son las habilidades empresariales más relevantes para enseñar? ¿Por qué?

5) ¿Cuáles son las habilidades empresariales menos relevantes para enseñar? ¿Por qué?

6) ¿Cree que las habilidades empresariales que enseña son útiles para el desarrollo profesional del alumnado? ¿Por qué?

7) ¿Aparte de estas habilidades, se podría impartir otro tipo de habilidades empresariales?

7.1) En caso afirmativo, ¿por qué? y ¿cuáles?

7.2) En caso negativo, ¿por qué no se puede impartir otro tipo de habilidades empresariales?

**Experiencia docente y habilidades empresariales de los programas de educación emprendedora**

Desde su experiencia como docente:

8) ¿Sería conveniente enseñar al alumnado todas las habilidades empresariales sobre el diseño de un proyecto/plan de empresa?

En caso negativo, ¿cuáles enseñaría? ¿Por qué?

En caso afirmativo, ¿por qué?

9) En el caso de que no tuviera tiempo para enseñar todas las habilidades empresariales del programa, ¿qué contenidos priorizaría para enseñar a sus alumnos? ¿Por qué?

10) Pensando en la Educación Secundaria Obligatoria como etapa educativa de formación empresarial básica, ¿qué habilidades empresariales enseñaría al alumnado para su posterior incorporación a la Formación Profesional o al Bachillerato?

11) ¿Cuáles son los criterios que utiliza para seleccionar y priorizar las habilidades empresariales destinadas al alumnado?

12) Pensando en su experiencia docente y en el contexto empresarial, ¿piensa que todas las habilidades empresariales del programa se adecuan a las necesidades formativas de las empresas? ¿Por qué?

**Cierre de la entrevista**

No tenemos más preguntas, aunque nos gustaría ofrecerle la oportunidad de añadir algún comentario u observación sobre las habilidades empresariales que son objeto de enseñanza, si así lo desea.

Le reiteramos nuestro agradecimiento por su participación.

**ENTREVISTA PARA ESTUDIANTES DE EDUCACIÓN SECUNDARIA PARTICIPANTES EN PROGRAMAS DE EDUCACIÓN EMPRESARIAL**

**Guía temática del entrevistador**

**Introducción a la entrevista**

En primer lugar, le expresamos nuestro agradecimiento por su participación.

Con esta entrevista pretendemos conocer su opinión, como alumno participante en programas de educación empresarial, sobre las habilidades empresariales que forman parte de los programas de este tipo de educación. Su valiosa información nos será útil para diseñar un instrumento de evaluación sobre habilidades empresariales básicas del alumnado, que forma parte de una amplia investigación sobre la educación empresarial en las etapas de la educación obligatoria.

Comentario sobre la confidencialidad:

Esta entrevista será grabada y posteriormente transcrita, garantizándole que todos los datos de la conversación serán confidenciales y anónimos. No existen respuestas correctas o incorrectas. Tiene total libertad para expresar su opinión y durante la entrevista puede solicitar la aclaración que precise. Con su permiso, vamos a comenzar a grabar.

**Inicio de la entrevista**

**Tipología de las habilidades empresariales en los programas de educación emprendedora**

Como estudiante:

1) ¿Qué habilidades empresariales se aprenden en el programa de educación emprendedora?

2) ¿Cuáles serían las habilidades empresariales necesarios para desarrollar un proyecto/plan de empresa?

Desde su punto de vista:

3) ¿Cuáles son las habilidades empresariales más importantes que ha aprendido? ¿Por qué?

4) ¿Cuáles son las habilidades empresariales menos importantes que ha aprendido? ¿Por qué?

Ha participado en programas de educación emprendedora en cursos anteriores, pensando en ellos:

5) ¿Qué clase de las habilidades empresariales aprendió en esos programas?

5.1) ¿Están vinculados a las habilidades empresariales que está aprendiendo en este curso académico?

5.2) ¿De qué modo?

5.3) ¿Podría poner ejemplos de la vinculación o relación de las habilidades empresariales de años anteriores con las actuales?

5.4) Considerando todo lo aprendido, ¿cómo clasificaría las habilidades empresariales adquiridas?

**Características de las habilidades empresariales en los programas de educación emprendedora**

Desde su punto de vista como estudiante:

6) ¿Qué son las habilidades empresariales? ¿Cómo los definiría?

7) De forma general, ¿Cómo son las habilidades empresariales?

Más concretamente:

7.1) ¿Están vinculados a la vida real?

7.2) ¿Cree que las habilidades empresariales aprendidas son aplicables de forma inmediata a la empresa? ¿Por qué? ¿Puede poner ejemplos o describir qué habilidades empresariales cree que son más aplicables y cuáles no?

7.3) ¿Quiere destacar alguna característica más de esas habilidades empresariales?

Comparando las habilidades empresariales del programa de educación empresarial de este curso académico con los programas de educación empresarial de cursos anteriores:

8) ¿Cuáles son las diferencias o similitudes de esas habilidades empresariales?

8.1) ¿Podría describir algunos ejemplos de las diferencias o similitudes?

9) Con las habilidades adquiridas, ¿se atrevería a iniciar un negocio? ¿Haría falta alguna habilidad no adquirida?

**Cierre de la entrevista**

No tenemos más preguntas, aunque nos gustaría ofrecerle la oportunidad de añadir algún comentario u observación sobre las habilidades empresariales que son objeto de enseñanza, si así lo desea.

Gracias por participar en esta entrevista.
