# Supplementary material for: Construction and validation of the Basic Scale of Entrepreneurial Competencies for the Secondary Education level. A study conducted in Spain
Source: PLoS One. 2021 Apr 15;16(4):e0249903. doi: 10.1371/journal.pone.0249903 (PMC8049328; doi:10.1371/journal.pone.0249903)
Supplement: S3 File — (DOC) [file pone.0249903.s004.doc]

**ESCALA BÁSICA DE HABILIDADES EMPRESARIALES PARA ESTUDIANTES DE EDUCACIÓN SECUNDARIA**

Lea cuidadosamente cada cuestión y marque su respuesta. Por favor, marque una sola respuesta en cada cuestión y responda a todas las cuestiones, según hayas sido capaz de hacer lo que indica cada ítem.

1= Nada; 2= Poco; 3= Suficiente; 4= Mucho

|  | ÍTEMS | 1 | 2 | 3 | 4 |
| --- | --- | --- | --- | --- | --- |
| 1 | Crear la marca publicitaria de una empresa. |  |  |  |  |
| 2 | Vender productos y/o servicios. |  |  |  |  |
| 3 | Diseñar un producto/servicio para una empresa. |  |  |  |  |
| 4 | Planificar y organizar la fabricación de productos/servicios en una empresa. |  |  |  |  |
| 5 | Fijar adecuadamente los precios de un producto/servicio. |  |  |  |  |
| 6 | Analizar las características de un producto/servicio. |  |  |  |  |
| 7 | Organizar a las personas en función del trabajo que vayan a realizar. |  |  |  |  |
| 8 | Elegir el modelo de gestión más adecuado para una empresa. |  |  |  |  |
| 9 | Diseñar el organigrama de una empresa. |  |  |  |  |
| 10 | Constituir una empresa y realizar los trámites para ejercer la actividad. |  |  |  |  |
| 11 | Elaborar el libro contable de una empresa. |  |  |  |  |
| 12 | Hacer una cuenta de resultados. |  |  |  |  |
| 13 | Calcular los costes, beneficios e ingresos de una empresa. |  |  |  |  |

Gracias por completar el cuestionario.
