# Supplementary material for: Construction and validation of the Basic Scale of Entrepreneurial Competencies for the Secondary Education level. A study conducted in Spain
Source: PLoS One. 2021 Apr 15;16(4):e0249903. doi: 10.1371/journal.pone.0249903 (PMC8049328; doi:10.1371/journal.pone.0249903)
Supplement: S4 File — (DOC) [file pone.0249903.s005.doc]

**BASIC SCALE OF ENTREPRENEURIAL COMPETENCIES FOR THE SECONDARY EDUCATION LEVEL**

Carefully read each question and mark your answer. Please check only one answer in each question and answer all questions. As you have been able to do what each item indicates.

1= Not at all; 2= Little; 3= Enough; 4 = A lot

|  | ÍTEMS | 1 | 2 | 3 | 4 |
| --- | --- | --- | --- | --- | --- |
| 1 | To create a firm’s brand advertising. |  |  |  |  |
| 2 | To sell products and/or services. |  |  |  |  |
| 3 | To design a product/service for a firm. |  |  |  |  |
| 4 | To plan and organize the manufacturing of products/services in a firm. |  |  |  |  |
| 5 | To appropriately set the prices of a product/service. |  |  |  |  |
| 6 | To analyze the characteristics of a product/service. |  |  |  |  |
| 7 | To organize people according to the work that they are going to do. |  |  |  |  |
| 8 | To choose the most appropriate management model for a firm. |  |  |  |  |
| 9 | To design a firm’s organigram. |  |  |  |  |
| 10 | To set up a firm and to carry out the procedures to practice the activity. |  |  |  |  |
| 11 | To elaborate a firm’s accounting book. |  |  |  |  |
| 12 | To do a results account. |  |  |  |  |
| 13 | To calculate a firm’s costs, profits and revenues. |  |  |  |  |

Thank you for completing the questionnaire.
